# Co-Culture with Two Soil Fungal Strains Enhances Growth and Secondary Metabolite Biosynthesis in *Cordyceps takaomontana*

**DOI:** 10.3390/jof11080559

**Published:** 2025-07-29

**Authors:** Junyi Chen, Minghao Ding, Donglan He, Dengxian Zhang, Ming Wang, Yulan Xiang, Tianya Liu

**Affiliations:** Hubei Provincial Engineering and Technology Research Center for Resources and Utilization of Microbiology, College of Life Science, South-Central Minzu University, Wuhan 430074, China

**Keywords:** *Cordyceps takaomontana*, soil microorganism, growth-promoting fungi, metabolomics

## Abstract

*Cordyceps takaomontana* is a medicinal fungus with significant pharmacological value, but how soil microbes promote its growth remains unclear. We established a solid-state co-culture system involving *C. takaomontana* synnemata and its native soil fungi of *Fusarium paeoniae* and *Bjerkandera minispora*. Both *F. paeoniae* and *B. minispora* significantly promoted synnematal growth and enhanced antioxidant enzyme activities. Total triterpenoid content increased substantially. *F. paeoniae* markedly elevated levels of ergosterol peroxide, whereas *B. minispora* boosted accumulation of L-arabinose, ergotamine, and euphol. Metabolomics revealed that both fungi activated key metabolic pathways (including ABC transporters, mineral absorption, and protein digestion/absorption). *F. paeoniae* uniquely upregulated phenylalanine metabolism. This work elucidates the metabolic mechanisms underlying growth promotion of *C. takaomontana* mediated by *F. paeoniae* and *B. minispora* as well as deciphers potential pharmacologically active metabolites. These findings provide a foundation for strategically improving artificial cultivation and developing functional microbial inoculants.

## 1. Introduction

Soil microecology refers to the dynamic interactive system formed between soil microorganisms and their physicochemical environment [1]. Within soil microecosystems, microbial interactions maintain the stability of material cycles among soil-dwelling organisms [2]. Consequently, elucidating the soil microbial interaction mechanisms in target species’ habitats constitutes a scientific prerequisite for developing conservation strategies. Among soil functional microorganisms, growth-promoting microorganisms directly enhance agricultural productivity [3]. In China, related studies predominantly focus on limited crop species, leaving vast reservoirs of potential growth-promoting microorganism resources unexplored [4,5]. Even when certain strains display growth-promoting effects, their host range remains notably narrow.

*Cordyceps takaomontana* Yakushiji & Kumazawa (1941), an entomopathogenic fungus parasitizing Lepidoptera insects, harbors multiple biological activities including improvement of glucose and lipid metabolism [6], and antithrombotic and antitumor effects [7,8]. As a member of the *Cordyceps* genus, this fungus typically has stringent environmental requirements, with its distribution constrained by multiple factors including climate, vegetation, and soil conditions [9,10]. Different *Cordyceps* species exhibit distinct environmental requirements, and environmental changes can significantly affect their yield [11]. Consequently, as reported in the literature, *C. takaomontana* and other *Cordyceps* species have limited distribution ranges. Currently, although artificial cultivation methods based on solid substrates (e.g., silkworm pupae and grains) have been developed for *C. takaomontana*, efficient approaches for its rapid growth remain insufficient [12,13]. Compared with the well-established cultivation systems for macrofungi like *Cordyceps militaris* [14], the cultivation techniques for this species are still in their infancy, urgently requiring more efficient growth methods to accelerate industrial-scale production. Given the paucity of research on environmental fungi affecting its growth, fungal regulation could serve as a potential novel approach for growth promotion. *Fusarium paeoniae*, a common species in Eurasian woodlands, has demonstrated plant growth-promoting and biocontrol functions in seedlings [15]. Although fungi from the *Bjerkandera* genus have shown growth-promoting effects in crops [16], their influence on *Cordyceps* remains unexplored.

Previous studies have identified potential plant growth-promoting microorganisms in *Cordyceps* habitats via functional prediction of microbial communities [17]. However, their growth-promoting characteristics remain theoretical without enough experimental evidence. An earlier study reports that isolated fungi of *Cordyceps sinensis* can enhance colony growth of *Cordyceps sinensis* by using plate co-culture experiments [18]. Nevertheless, reports on fungal effects on *Cordyceps* synnemata remain remarkably scarce. To systematically elucidate the impact of soil fungi on *C. takaomontana* growth, it is essential to establish both a co-culture system with *Cordyceps* synnemata and adopt comprehensive analytical techniques to identify bioactive components. Conventional high-performance liquid chromatography (HPLC) with an external standard method is primarily employed for analyzing common compounds in *Cordyceps* fungi, including ergosterol, cordycepin, and so on [19,20]. In contrast, untargeted metabolomics (UM) frequently identifies previously unreported metabolites that are closely associated with specific biological activities in this kind of fungal genus [21,22]. Riding on the fast development of UM, it enables us to better understand metabolic characteristics of *Cordyceps* fungi under conditions with or without beneficial fungi.

To unveil the knowledge gap mentioned above, we aimed to (i) isolate growth-promoting fungi from *C. takaomontana* habitat soil; (ii) establish the co-culture system between two isolated growth-promoting fungi and *C. takaomontana* synnemata; (iii) decipher responses of *C. takaomontana* to the inoculation of *F. paeoniae* or *B. minispora* by monitoring physio-biochemical changes in synnemata pre- and post-inoculation; and (iv) identify potential active metabolites and reveal metabolic pathways via UM. Here, we hypothesized that the fungi isolated from *C. takaomontana* habitat soil would greatly enhance fungal growth and benefit compound production. These findings provide a scientific foundation for sustainable resource utilization and cultivation system optimization for *Cordyceps* fungi, and this study reports a candidate of functional microbial inoculant for *Cordyceps* fungi.

## 2. Materials and Methods

### 2.1. Acquisition of C. takaomontana and Its Culture Media

Wild *C. takaomontana* were collected from Hongping Town, Shennongjia Forestry District, Hubei Province, China (110°21′, 31°34′). Pure mycelia were obtained via tissue isolation method and deposited at the Hubei Provincial Engineering Research Center for Microbial Resources and Utilization, School of Life Sciences, South-Central Minzu University (114°23′, 30°29′). The culture media include (1) Sabouraud solid medium (g/L): glucose 40.00, yeast extract 10.00, soy peptone 10.00, agar 20.00; (2) Sabouraud liquid medium: identical composition without agar; (3) *Cordyceps*-specific nutrient solution (g/L): sucrose 20.00, peptone 5.00, MgSO_4_ 1.00, KH_2_PO_4_ 1.00; (4) cultivation substrate: sterilized wheat grains (20 g/bottle). For metabolomic analysis, a UPLC column (2.1 mm × 100 mm, 1.7 μm; Waters, Milford, MA, USA) with methanol/acetonitrile mobile phase (HPLC grade; Macklin, Tokyo, Japan) was employed.

### 2.2. Isolation and Identification of Soil Fungi

Surface soil samples (0–5 cm depth) were randomly collected from four points within 5 cm of *C. takaomontana* growth sites, and these soils were designated as habitat soil of *C. takaomontana*. Fungi were isolated by grinding soil in sterile water, homogenizing, and performing 10-fold serial dilutions. We took 1 mL of the suspension and spread it on Potato Dextrose Agar (PDA) plates supplemented with 50 mg/mL each of streptomycin and ampicillin. After 5-day incubation at 26 °C, emergent colonies were transferred to fresh solid PDA for purification. We selected two isolates with different morphology and designated them as S1 and S2. Colony morphology was documented, with microscopic examination using lactophenol cotton blue staining. Colony and spore morphology were documented, with spore dimensions measured using an ocular micrometer calibrated to 1 μm per division at 100× magnification.

Genomic DNA was extracted from S1/S2 using the Solarbio Fungal DNA Kit (Beijing, China). The internal transcribed spacer (ITS) region was amplified by using universal primers ITS1 (5′-TCCGTAGGTGAACCTGCGG-3′) and ITS4 (5′-TCCTCCGCTTATTGATATGC-3′). Sequences were assembled/trimmed in ContigExpress (v1.0) and then subjected to BLASTn (v2.13) against NCBI Nucleotide (www.ncbi.nlm.nih.gov) for homology identification and accession number acquisition. Phylogenetic trees were constructed by applying MEGA (v11.0.13) software based on neighbor-joining with 1000 bootstrap replicates.

### 2.3. Growth-Promoting Traits of Soil Fungi

Through biochemical assays, the growth-promoting traits of strains S1 and S2 were evaluated, including quantification of Indole-3-acetic acid (IAA) content, siderophore production, nitrogen-fixing activity, and potassium-solubilizing capacity. IAA content was determined using the Salkowski method [23]. Siderophore production was assessed via the Chrome Azurol S (CAS) assay [24]. Nitrogen-fixing ability was tested in Ashby’s nitrogen-free medium [25]. Potassium-solubilizing characteristics were examined in potassium feldspar medium [26]. This experiment was biologically repeated three times.

### 2.4. Artificial Cultivation of C. takaomontana and Establishment of Co-Culture System

Each culture bottle was filled with cultivation substrate and supplemented with 30 mL of nutrient solution, followed by incubation at 16 °C and 60% relative humidity in a constant-temperature biochemical incubator under dark conditions. After full mycelial colonization of the substrate, cultures were transferred to light-incubation chambers. Primordia differentiation conditions were maintained at 20 °C and 65% RH under photoperiod control, with 12 h white light (750 lux) exposure and 12 h darkness cycles.

Fungal plugs (6 mm diameter) were aseptically excised from S1/S2 PDA cultures using a sterile cork borer and transferred into 250 mL Erlenmeyer flasks containing 100 mL of Potato Dextrose Broth (PDB), followed by 5-day incubation at 26 °C with shaking (160 rpm). Mycelial pellets were homogenized, centrifuged at low speed, washed with sterile water, and resuspended to yield 2 g (wet weight) inoculum. Upon primordium formation and synnemata emergence in *C. takaomontana* culture bottles, vessels exhibiting uniform fungal distribution and growth were selected for inoculation. Experimental groups (S1/S2) received 2 mL of respective fungal suspensions, while the control group (designated CK hereafter) was administered equal volumes of sterile water. All cultures were maintained at 26 °C and 65% relative humidity in constant-temperature biochemical incubators under white light exposure (750 lux). After 25 days post-inoculation, synnematal count, height, and fresh weight per bottle were quantified.

### 2.5. Quantification of Antioxidant Active Constituents in Cordyceps takaomontana

To preliminarily determine bioactive compounds in *C. takaomontana*, aqueous and methanol extracts were prepared. Synnemata were oven-dried at 60 °C, pulverized, and then subjected to ultrasonic extraction (1:10 *w*/*v*) at 50 °C (aqueous) or 30 °C (methanol) for 4 h, followed by agitation (130 rpm) for 24 h. Total phenolic content in aqueous extracts was quantified using the Folin–Ciocalteu method using gallic acid calibration standards (0.01–0.06 mg/L), with synnemata results expressed as gallic acid equivalents [27]. Flavonoid content was determined via aluminum chloride colorimetry with rutin standards (0.01–0.06 mg/mL), reported as rutin equivalents [28]. Total triterpenoids in methanol extracts were analyzed by vanillin–acetic acid assay using oleanolic acid standards (0.02–0.10 mg/L), calculated as oleanolic acid equivalents [29]. All bioactive constituents were quantified in mg/g dry weight. This experiment was biologically repeated three times.

### 2.6. Antioxidant Enzyme Activity Assay of Cordyceps takaomontana

To evaluate antioxidant enzyme activities, aqueous extracts were centrifuged at 12,000 rpm and 4 °C for 10 min, followed by supernatant collection for enzymatic assays. Peroxidase (POD)-like activity was assessed via guaiacol colorimetry at 470 nm, with one unit (U) defined as the amount catalyzing 1 μg substrate conversion per mg tissue per minute. Superoxide dismutase (SOD)-like activity was determined by nitroblue tetrazolium (NBT) reduction assay, where one unit (U) represented the enzyme quantity inhibiting 50% NBT reduction in 1 mL reaction system [30]. Catalase (CAT)-like activity was measured using ammonium molybdate colorimetry at 405 nm, monitoring absorbance changes of the yellow complex [31]. All assays were performed in three biological replicates.

### 2.7. Sample Preparation and Implementation of Untargeted Metabolomics

To analyze metabolic impacts of strains S1/S2 on *C. takaomontana* synnemata, *Cordyceps* analytical samples were dissolved in methanol/acetonitrile/water (2:2:1, *v*/*v*), vortex-mixed, and ultrasonicated at 4 °C for 30 min. After centrifugation (14,000 rpm, 4 °C, 20 min), supernatants were vacuum-dried. Residues were reconstituted in 100 μL acetonitrile/water (1:1, *v*/*v*), vortexed, and recentrifuged (14,000 rpm, 4 °C, 15 min) prior to LC-MS analysis.

The supernatants after secondary centrifugation, with each group comprising six biological replicates, were loaded into a 4 °C autosampler and separated on an Agilent 1290 UHPLC system equipped with a C_18_ column (2.1 mm × 100 mm, 1.7 μm, Waters, Milford, MA, USA). Mobile phases comprised (A) water with 25 mmol/L ammonium acetate and 0.5% formic acid, (B) methanol. Gradient elution (Table 1) was applied at 0.4 mL/min and 40 °C with 2 μL injection volume. Throughout the analytical process, samples were maintained at 4 °C in the autosampler. Quality control samples were interspersed to monitor system stability.

Mass spectrometric data acquisition was performed on an AB Triple TOF 6600 system (SCIEX, Redwood, CA, USA), collecting both primary (MS1) and secondary (MS2) spectra. Electrospray ionization (ESI) conditions were set as follows: Ion Source Gas 1 (GS1) 60 psi, Ion Source Gas 2 (GS2) 60 psi, Curtain Gas (CUR) 30 psi, source temperature 600 °C, Ion Spray Voltage Floating (ISVF) ±5500 V for both polarities; TOF MS scan range *m*/*z* 60–1000 Da, product ion scan range *m*/*z* 25–1000 Da, accumulation times 0.20 s/spectrum for MS and 0.05 s/spectrum for MS/MS; MS/MS spectra were acquired via information-dependent acquisition (IDA) in high-sensitivity mode with declustering potential (DP) ±60 V and collision energy 35 ± 15 eV, using IDA settings that excluded isotopes within 4 Da and monitored 10 candidate ions per cycle.

### 2.8. Data Analysis

Growth parameters of *Cordyceps* synnemata and their antioxidant activity data were analyzed by analysis of variance (ANOVA) followed by multiple comparison tests. Statistical analyses were performed using GraphPad Prism 8.0. For non-targeted metabolomics, Principal Component Analysis (PCA) was separately applied to positive and negative ion mode datasets from all *Cordyceps* samples for quality assessment. Then, the data were analyzed through univariate and multivariate statistical analyses, differential metabolite screening, and Kyoto Encyclopedia of Genes and Genomes (KEGG) pathway analysis.

## 3. Results

### 3.1. Colony Characteristics and Species Identification of Isolated Fungi

**Figure 1:**
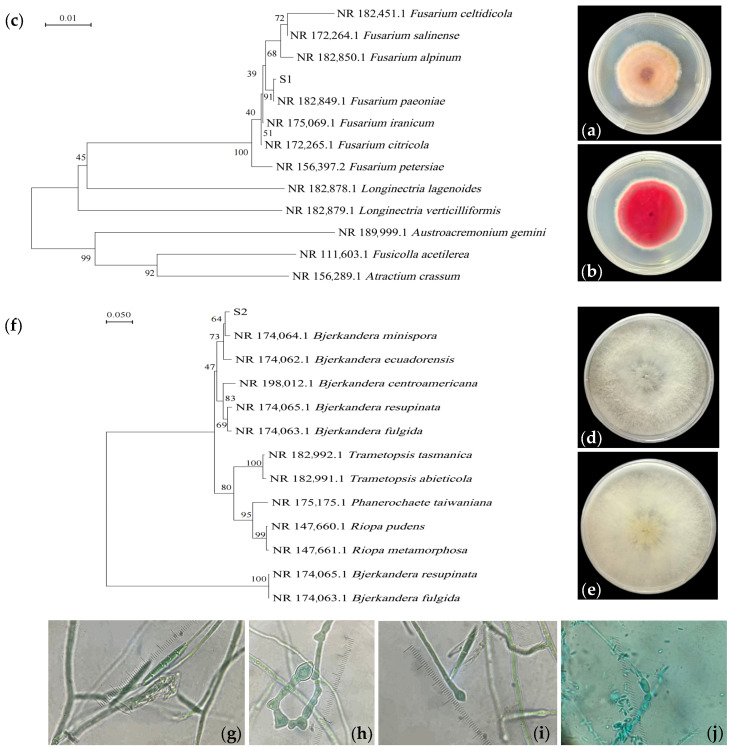
Morphological and molecular characteristics of isolated fungi. (**a**,**b**) Morphology of strain S1 colonies; (**c**) phylogenetic neighboring tree of strain S1 based on ITS by using neighbor-joining method with 1000 bootstrap replicates; (**d**,**e**) morphology of colony S2 colonies; (**f**) phylogenetic neighboring tree of strain S2 based on ITS by using neighbor-joining method with 1000 bootstrap replicates; (**g**) conidiophores (100×); (**h**,**i**) thick-walled spores (100×); (**j**) spores of strain S2 (100×).

After 7 days of growth on PDA medium, strain S1 exhibited orange-yellow colonies with radial patterns, featuring villous surfaces and aerial hyphae on the obverse side, while the reverse side showed rose-red pigmentation (Figure 1a,b). The conidia were fusiform with three septa (Figure 1g), and chlamydospores appeared elliptical, either terminal or intercalary (Figure 1h,i). Strain S2 formed white villous colonies with fluffy aerial growth and loose texture, lacking pigmentation on both obverse and reverse sides (Figure 1d,e). Its conidia were clavate (Figure 1j). Based on morphological characteristics, strains S1 and S2 were preliminarily identified as *Fusarium* sp. and *Bjerkandera* sp., respectively.

Phylogenetic trees were constructed using the neighbor-joining method based on homologous sequences. The results demonstrated that strain S1 (accession number: PV919832) clustered with *Fusarium paeoniae* with 99.58% similarity (Figure 1c), while strain S2 (accession number: PV919833) showed 97.78% similarity to *Bjerkandera minispora* (Figure 1f). Consequently, strain S1 was identified as *F. paeoniae*, and strain S2 as *B. minispora*.

### 3.2. Growth-Promoting Traits of Strains

Both strains exhibited IAA biosynthesis capacity, with S1 demonstrating superior performance (55.739 μg/mL). Qualitative assays for nitrogen fixation and siderophore production suggested that both strains may facilitate soil mineral/organic matter mobilization through biological nitrogen fixation and organic ligand secretion. However, neither strain displayed potassium-solubilizing activity on K-feldspar medium. Collectively, these traits indicated their potential to influence the growth of *C. takaomontana* (Table 2).

### 3.3. Physiological Indices of C. takaomontana

**Figure 2:**
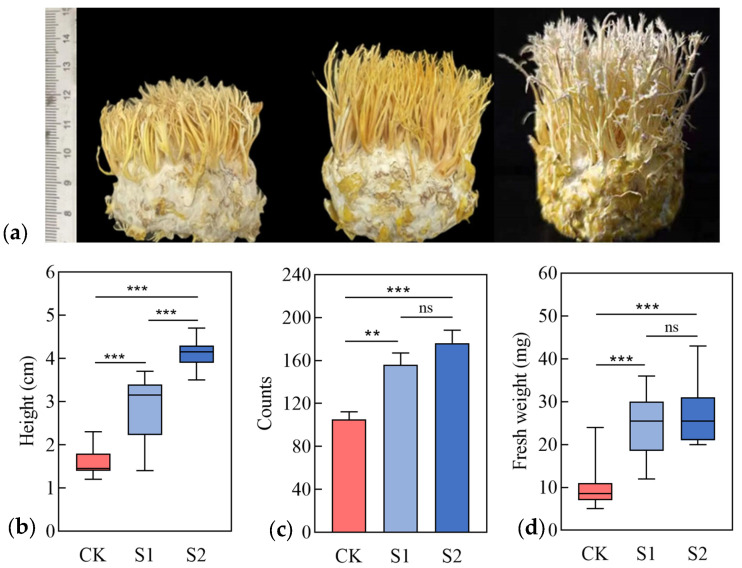
Growth promotion potential of isolated fungi on *C. takaomontana* synnemata. (**a**) Morphological characteristics of synnemata in *C. takaomontana.* Boxplots display differences in height (**b**) and coremia counts fresh weight of *C. takaomontana* among three groups (each box contains 21 replicates) (**c**). Fresh weight shows the difference in coremia counts among three groups (**d**). The results are the mean values, and error bars denote standard deviation. Asterisks in panels (i.e., (**b**–**d**)) represent significance (**, *p* < 0.01; ***, *p* < 0.001). ‘ns’ denotes absence of significant differences.

After 25 days of co-cultivation with strains S1 and S2, the synnemata of *C. takaomontana* inoculated with either strain S1 or S2 exhibited faster growth rates compared to the control group (CK). The growth-promoting effect was more pronounced in the S2 group than in the CK group. The S2-induced *C. takaomontana* displayed the fastest growth, with the apex developing snowflake-like white spores (Figure 2a). The average height of synnemata in the S2 group reached 4.1 cm, representing 1.44 times that of the S1 group (*p* < 0.0001) and 2.56 times that of the CK group (*p* < 0.0001) (Figure 2b). The average numbers of synnemata in the S1 and S2 groups were 1.48-fold and 1.67-fold higher than the CK group, respectively (Figure 2c). Although there was no significant difference in the number of synnemata or fresh weight between the S1 and S2 groups, morphologically, the synnemata in the S1 group were the thickest and exhibited the highest fresh weight, averaging 24.55 mg (Figure 2d). These results demonstrated that both *F. paeoniae* and *B. minispora* significantly promoted the growth of *C. takaomontana*.

### 3.4. Antioxidant Activity of C. takaomontana

**Figure 3:**
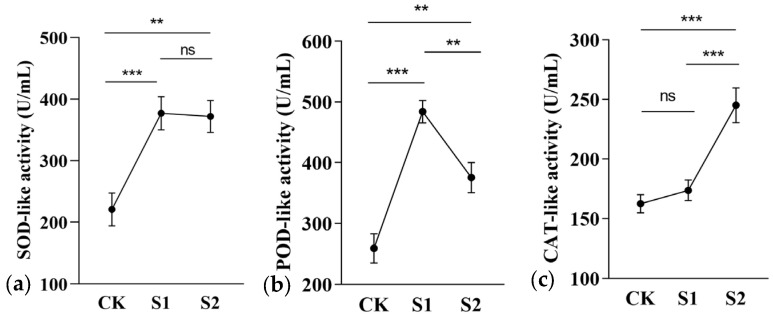
Changes in antioxidant enzyme activities of *C. takaomontana* synnemata. (**a**) SOD-like activity; (**b**) POD-like activity; (**c**) CAT-like activity. The results are the mean values of three replicates, and error bars denote standard deviation. Asterisks in panels (i.e., (**a**–**c**)) represent significance (**, *p* < 0.01; ***, *p* < 0.001), ‘ns’ denotes absence of significant differences.

**Figure 4:**
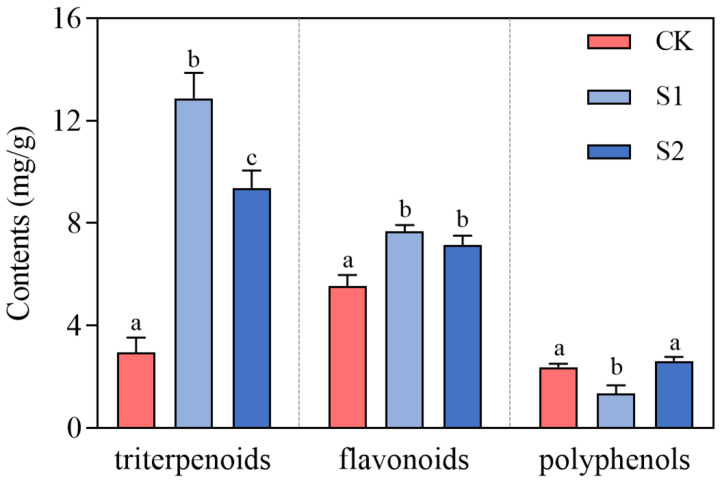
Antioxidant compound contents of *C. takaomontana* synnemata. The results are the mean values of three replicates, and error bars denote standard deviation. Different lowercase letters above columns represent significance (*p* < 0.05).

The results indicated that the experimental groups enhanced the activities of three antioxidant-like enzymes in *C. takaomontana*, demonstrating that soil microbial treatment effectively activated its antioxidant defense system. There was no significant difference between the S1 and S2 groups in the magnitude of increase in SOD-like enzyme activity (Figure 3a). The S1 group treatment showed the greatest increase in POD enzyme activity (Figure 3b), while the S2 group treatment showed the greatest increase in CAT enzyme activity (Figure 3c). Regarding antioxidant compounds, both treatments significantly increased total triterpenoid accumulation in synnemata (S1 group showed the highest efficacy). While total flavonoids displayed an increasing trend, no significant difference was observed between S1 and S2 groups in flavonoid enhancement. However, strain S1 significantly reduced total phenolic content, which may be associated with differential regulation of its metabolic pathways (Figure 4). These data suggested that soil fungi S1 and S2 activated stress resistance mechanisms in *C. takaomontana* synnemata by modulating its antioxidant system.

### 3.5. Non-Targeted Metabolomic Analysis

#### 3.5.1. Quality Assessment of the Metabolic Model

**Figure 5:**
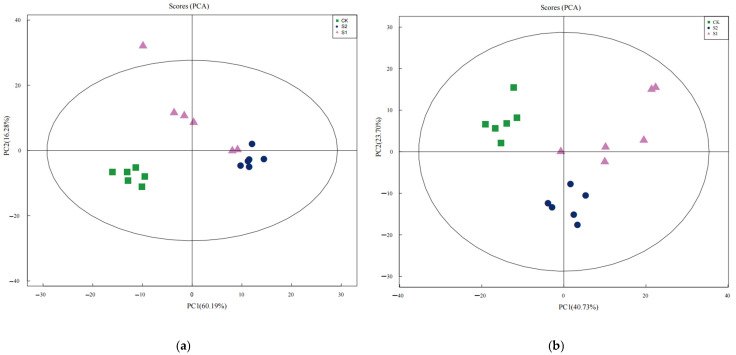
PCA plots showing compositional similarity of metabolites using positive ion model (**a**) and negative ion model (**b**).

To ensure the reliability of the metabolomics data, PCA was employed to evaluate differences between sample groups. In the constructed PCA model, samples from the CK, S1, and S2 groups were represented by green, red, and blue dots, respectively, with each dot denoting an individual biological replicate. The eigenvector accounting for the maximum explained variance was designated as the first principal component (PC1), while the eigenvector with the second-largest explained variance constituted the second principal component (PC2). In positive ion mode, PC1 and PC2 collectively explained 76.47% of the total data variability (Figure 5a); in negative ion mode, they accounted for 64.03% of the overall variability (Figure 5b). Both values exceeded the 60% threshold. In both positive and negative ion modes, the three sample groups exhibited clear separation within the principal component space, indicating their distinctive metabolic profiles. The pronounced divergence between the CK group and the experimental groups highlighted the significant impact of soil fungal inoculation on the metabolite composition of *C. takaomontana*. Furthermore, the clear separation between the S1 and S2 groups suggested strain-specific regulatory effects of the environmental microorganisms on *C. takaomontana*. The tight clustering of intragroup biological replicates further validated the operational stability throughout the entire process, from sample preparation to LC-MS analysis, and confirmed data reliability.

#### 3.5.2. Analysis of Metabolite Composition

**Figure 6:**
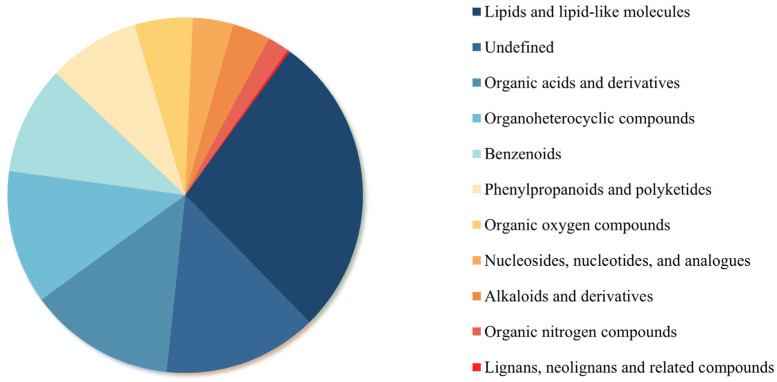
Pie chart showing percentages of different metabolite types.

Metabolite composition was profiled using a UPLC-MS platform. Based on the KEGG metabolic database, 871 metabolites were identified and categorized into 11 distinct superclasses (Figure 6). Lipids and lipid-like molecules constituted the predominant metabolic component, accounting for 27.6% of the total metabolites. This was followed by uncharacterized compounds (14.05%), organic acids and derivatives (13.32%), and organoheterocyclic compounds (10.00%). Organic oxygen compounds represented 5.00%. In contrast, the least abundant category was lignans, neolignans, and related compounds, comprising only 0.23% of the total metabolites.

#### 3.5.3. Analysis of Differential Metabolites

**Figure 7:**
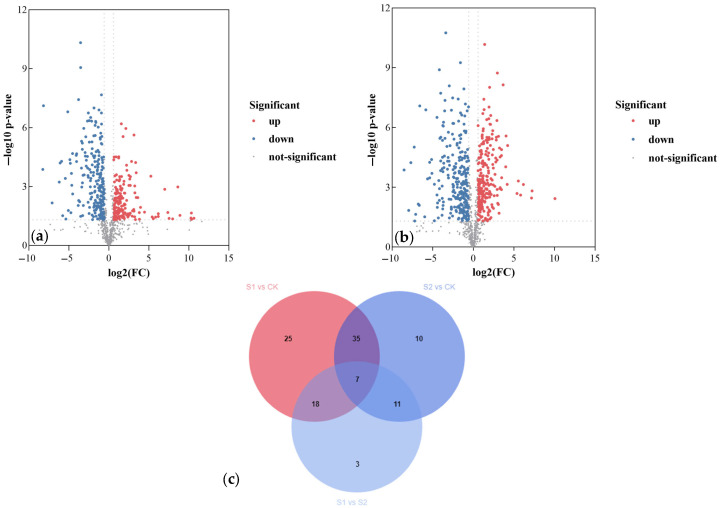
Analysis of differential metabolite abundance. (**a**) Volcano plots comparing the S1 and CK groups; (**b**) Volcano plots comparing the S2 and CK groups; (**c**) Venn diagram of differential metabolites across the three groups.

Volcano plot analysis revealed 412 and 504 differential metabolites in the S1 and S2 groups, respectively, compared to the CK group, indicating that both treatments induced significant alterations in metabolic profiles. Specifically, the S1 group exhibited 188 upregulated and 224 downregulated metabolites (Figure 7a), while the S2 group showed 244 upregulated and 260 downregulated metabolites (Figure 7b).

Metabolites in the S1 group meeting the criteria of FC > 1.2 or <0.8 with VIP ≥ 1 are listed in Supplementary Table S1, with the top 30 ranked differential metabolites visualized using bar plots. Compared to the CK group, the S1 group exhibited significantly increased levels of terpenoids and sterols, with remarkably higher accumulation of notoginsenoside R1 (FC: 3179.87) and ergosterol peroxide (FC: 10.71) (Figure S1). Upregulated metabolites primarily belonged to organic acids and derivatives, including enniatin B (FC: 1606.94), pantothenic acid (FC: 2.55), glutamine (FC: 1.78), and arginine (FC: 1.61). Additionally, pharmacologically active components such as L-carnitine (FC: 1.98) and cinnamic acid (FC: 1.64) were upregulated. Conversely, downregulated metabolites were mainly fatty acids, with trans-vaccenic acid exhibiting the highest variable importance in projection (VIP) value (VIP: 9.94). Other downregulated metabolites included carboxylic acids and derivatives, such as proline (FC: 0.65), cyclo(VVFF) (FC: 0.29), and betaine (FC: 0.58). Furthermore, D-glucose was found to be downregulated.

Supplementary Table S2 lists S2-group metabolites meeting the criteria (FC > 1.2 or <0.8; VIP ≥ 1), with bar plots visualizing the top 30 ranked differential metabolites (Figure S2). In the S2 group, Ergosterol peroxide (FC: 10.99) and euphol (FC: 3.60), along with terpenoids, were increased. Organic oxygen compounds were also upregulated, with L-arabitol showing significant upregulation (FC: 16.34), followed by sorbitol-6-phosphate (FC: 2.62) and trehalose-6-phosphate (FC: 2.14), suggesting activation of the pentose phosphate pathway. Concurrently, increases were observed in organic oxygen compounds such as cyclo(valylprolyl) (FC: 2.06) and glutamine (FC: 1.54). Additionally, the production of pharmacologically active substances, including p-coumaraldehyde (FC: 5.26) and ergotamine (FC: 2.29), was enhanced. Conversely, downregulated metabolites included D-glucose, glucose-6-phosphate, the phenolic compound gingerol, and the flavonoid peonidin-3,5-O-di-β-glucopyranoside. These metabolites may represent key factors influencing the growth.

Furthermore, Venn diagram analysis identified 43 and 21 unique differential metabolites specific to the S1 and S2 groups compared to the control, respectively, implying distinct mechanisms by which the two strains promote *C. takaomontana* growth. Additionally, 42 differential metabolites were common to both the S1 and S2 groups when compared to the CK group, with 7 metabolites shared among all three groups (Figure 7c).

#### 3.5.4. Analysis of Differential Metabolic Pathways

**Figure 8:**
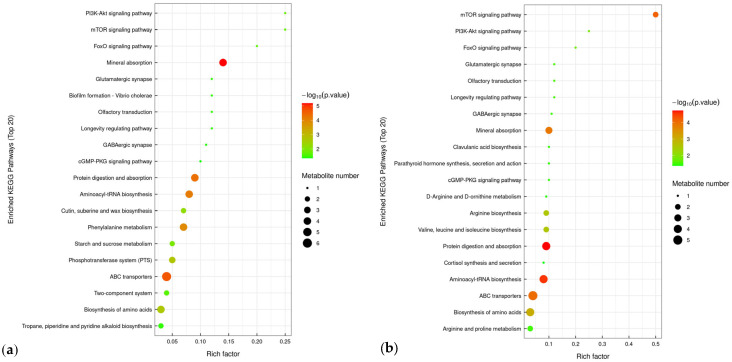
KEGG pathway enrichment analysis of differential metabolites. (**a**) S1 group and CK group; (**b**) S2 group and CK group. Bubble size represents the metabolite number; color gradient indicates the significance level of enrichment.

KEGG pathway enrichment analysis revealed that differential metabolites in the S1 and S2 groups were significantly enriched in ABC transporters, mineral absorption, protein digestion and absorption, and aminoacyl-tRNA biosynthesis pathways (Figure 8). The enhanced activity of ABC transporter pathways suggested that strains S1 and S2 promoted active transmembrane transport in *C. takaomontana*. Enrichment of mineral and protein metabolic pathways indicated that soil fungi S1 and S2 may drive growth and metabolism in *C. takaomontana* by regulating metal ion transport and improving nitrogen source utilization efficiency. Furthermore, strain S1 specifically exhibited enrichment of differential metabolites in phenylalanine metabolism and phosphotransferase system pathways.

## 4. Discussion

### 4.1. Inoculation of Isolated Fungi Promoting the Growth of C. takaomontana

This study, to our knowledge, is the first to successfully promote the growth of *C. takaomontana* synnemata by using microbial inoculates of *F. paeoniae* or *B. minispora*. Under identical solid cultivation substrate conditions, the inoculation of *F. paeoniae* and *B. minispora* demonstrated a more significant extent of promotion in synnemata height compared to nutritional supplementation [12]. However, this study has not yet optimized individual factors influencing fungal growth, particularly the inoculation volume of growth-promoting bacterial agents. The importance of this factor has been confirmed in previous studies on promoting the colonial growth of *C. sinensis* [18].

### 4.2. Differential Metabolic Pathways

This study reported the first detection of trehalose-6-phosphate (T6P) in *C. takaomontana*. As an established phytohormone, T6P exhibited growth-promoting effects consistent with prior research [32], suggesting its potential role in regulating *Cordyceps* synnemata morphogenesis, though further mechanistic investigations are required for validation. Under *B. minispora* induction, T6P content significantly increased in synnemata, likely due to activation of trehalose biosynthesis—a reserve carbohydrate degraded under specific conditions in macrofungi that served as a carbon source translocated from mycelia to fruiting bodies [33]. Concurrently, both soil fungi activated sugar alcohol synthesis pathways (e.g., arabitol, mannitol), which may function as osmoprotectants during synnematal development.

Amino acids demonstrated potent metal-chelating capacity for immobilizing soil ions [34]. *F. paeoniae* synergistically activated amino acid metabolism and mineral absorption pathways in *C. takaomontana*, leading to significant accumulation of glutamine, arginine, and their derivatives. We propose a plausible mechanism whereby amino acids may enhance mineral uptake in *C. takaomontana* through chelation, thereby promoting fungal growth. This hypothesis requires future validation using atomic absorption spectroscopy (AAS). Furthermore, *B. minispora* induced phenylalanine metabolism—critical for nitrogen allocation in hosts [35]—though how phenylalanine modulates nitrogen supply during development requires further investigation.

The *Cordyceps* readily accumulated heavy metals [36,37], which positively regulated mycelial biomass. Soil fungi activated mineral absorption pathways, corroborating this growth-promotion mechanism. Although both soil fungal strains possessed siderophore-producing capabilities, the causal relationship between this trait and differential metabolic pathways requires further experimental validation through isotope labeling techniques.

### 4.3. Fungal-Induced Antioxidant Defense Activation

*F. paeoniae* and *B. minispora* significantly enhanced antioxidant capacity in *C. takaomontana* synnemata, indicating imposed biotic stress. The host activated its antioxidant defense system against oxidative damage, evidenced by elevated activities of antioxidant enzymes (e.g., POD, CAT, SOD) and accumulation of non-enzymatic antioxidants (e.g., total flavonoids). While most antioxidant indicators increased under fungal induction, total phenolic content decreased specifically with *B. minispora*—a phenomenon requiring further analysis. Furthermore, non-enzymatic antioxidants such as flavonoids, glutathione, and proline play critical roles in host growth and development [38]. In this study, glutathione and proline were identified as differential metabolites, potentially explaining why enhanced antioxidant activity frequently correlates with accelerated growth rates in hosts across numerous studies [39].

### 4.4. Enhancement of Beneficial Secondary Metabolites

Biotic stress serves as a critical driver for the accumulation of secondary metabolites and quality in host organisms. By activating biosynthetic pathways, it enhanced endogenous secondary metabolite levels and bolstered stress resistance, thereby ensuring host growth [40]. Utilizing untargeted metabolomics, we identified multiple bioactive compounds in *C. takaomontana* that have not been previously reported within the *Cordyceps* genus. LC-MS confirmed notoginsenoside R_1_ in synnemata—overcoming the limitation of colorimetric methods in prior studies that could only detect total terpenoids without resolving specific compositions [41]—marking its first report in *Cordyceps* to our knowledge. This saponin, typically found in Araliaceae plants (*Panax* spp.), was renowned for its hemostatic/anti-inflammatory/cardioprotective properties [42,43,44]. Under *F. paeoniae* induction, notoginsenoside R_1_ content increased 3179-fold (FC = 3179)—the highest fold-change among differential metabolites. This demonstrated that soil fungi regulate saponin accumulation in the host, aligning with an existing report [45]. Furthermore, significantly altered metabolites included multiple antibacterial substances (e.g., enniatin B [46], benzaldehyde [47] (with broad-spectrum antimicrobial activity)), suggesting that the soil fungi *F. paeoniae* and *B. minispora* may regulate the antimicrobial pharmacological activity of *C. takaomontana*. However, their specific efficacy requires further validation through In vitro pharmacological assays on synnemata. Ergosterol peroxide—a sterol with antiviral/anti-inflammatory [48,49] and antitumor properties [50]—was enriched by both fungi, though activation mechanisms require further study. Euphol, a steroidal compound rarely documented in *Cordyceps* fungi, is renowned for its anti-inflammatory/anticancer efficacy in breast/prostate cancer cell lines [51,52,53,54]. Visnagin—a co-downregulated metabolite in both S1 and S2 groups—possessed anti-inflammatory [55] and anti-diabetic properties [56]. These findings revealed the regulatory role of soil fungi in the biosynthesis of medicinal compounds in the *Cordyceps*, paving the way for targeted synthesis strategies of these bioactive metabolites.

### 4.5. Study Limitations and Future Work

This study has several limitations that warrant improvement: (1) While we have established the growth-promoting effects of two fungal species on *C. takaomontana*, the underlying mechanism—whether mediated through symbiotic interactions or direct fungal metabolites—remains unclear. Future investigations should employ scanning electron microscopy to examine symbiotic structures and conduct independent metabolite functional assays for verification; (2) the simplified single-strain-*Cordyceps* model cannot fully recapitulate the complex interactions occurring in natural soil microbial communities; (3) isotope labeling experiments should be implemented to elucidate mineral element-mediated growth promotion mechanisms.

## 5. Conclusions

This study firstly demonstrated the growth-promoting effects of habitat soil fungi *Fusarium paeoniae* and *Bjerkandera minispora* on *Cordyceps takaomontana*. Both fungi significantly increased its biomass and activated antioxidant defense systems, with metabolic pathway enrichment analysis revealing key regulations in mineral absorption, amino acid biosynthesis, and ABC transporters. *F. paeoniae* specifically enhanced the phenylalanine metabolism and phosphotransferase system pathways. Untargeted metabolomics identified pharmacologically active compounds including notoginsenoside R_1_, euphol, and ergotamine in *C. takaomontana*. Notably, notoginsenoside R_1_ accumulation was elevated 3179-fold under *F. paeoniae* treatment, highlighting this species as a sustainable source of bioactive metabolites. These findings provided novel insights into the functional mechanisms of soil-derived fungi on the *Cordyceps* genus, offering significant value for deciphering ecological adaptations and exploiting natural products.

## Figures and Tables

**Table 1:** Gradient elution program.

Time (min)	A/%	B/%
0–0.5 min	95	5
0.5–12.0 min	0	100
12.0–12.1 min	95	5
12.1–16.0 min	95	5

**Table 2:** Growth-promoting effect of strains S1 and S2.

Growth-Promoting Traits	Fungus S1	Fungus S2
IAA Production (μg/mL)	55.739	46.856
Potassium Solubilization	n.a.	n.a.
Siderophore Production	+	+
Nitrogen Fixation	+	+

“n.a.” indicates no activity detected, and “+” indicates positive activity.

## Data Availability

The original contributions presented in this study are included in the article and Supplementary Materials. Further inquiries can be directed to the corresponding author.

## Supplementary Materials

The following supporting information can be downloaded at: https://www.mdpi.com/article/10.3390/jof11080559/s1, Table S1: Differential metabolites in the S1 group (FC > 1.2 or < 0.8 and VIP ≥ 1); Table S2: Differential metabolites in the S2 group (FC > 1.2 or < 0.8 and VIP ≥ 1); Figure S1: The top 30 differential metabolites ranked by FC values between S1 and CK groups. Metabolites with significant up-regulation were shown in red; significant down regulation were shown in blue; Figure S2: The top 30 differential metabolites ranked by FC values between S2 and CK groups. Metabolites with significant up-regulation were shown in red; significant down regulation were shown in blue.
