# Supplementary material for: Co-Culture with Two Soil Fungal Strains Enhances Growth and Secondary Metabolite Biosynthesis in Cordyceps takaomontana
Source: J Fungi (Basel). 2025 Jul 29;11(8):559. doi: 10.3390/jof11080559 (PMC12387924; doi:10.3390/jof11080559)
Supplement: Supplementary file 1 [file jof-11-00559-s001.zip › Supplementary Materials.pdf]

Supplementary materials

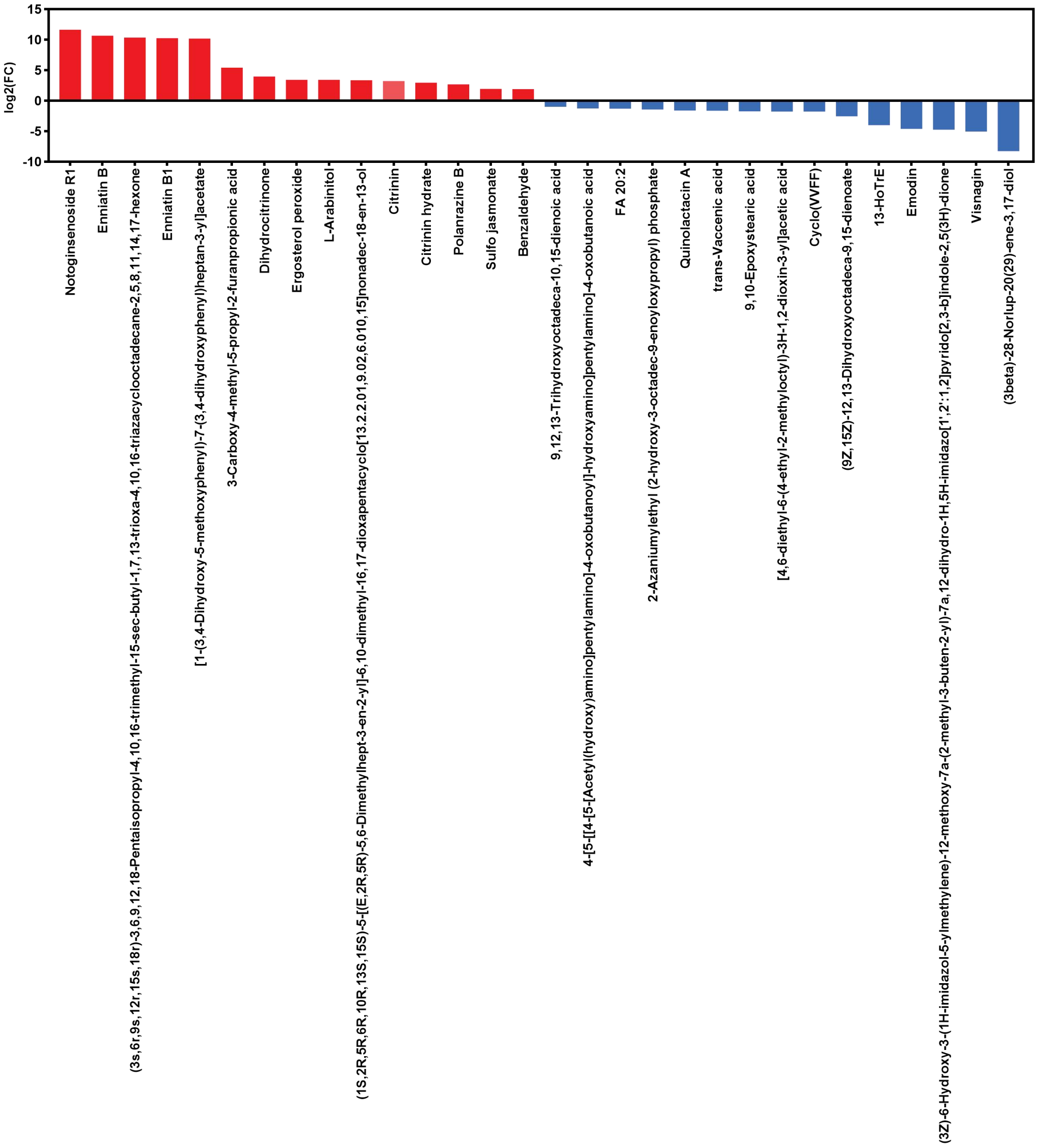

Figure S1. The top 30 differential metabolites ranked by FC values between S1 and CK groups. Metabolites with significant up-regulation were shown in red; significant down-regulation were shown in blue.

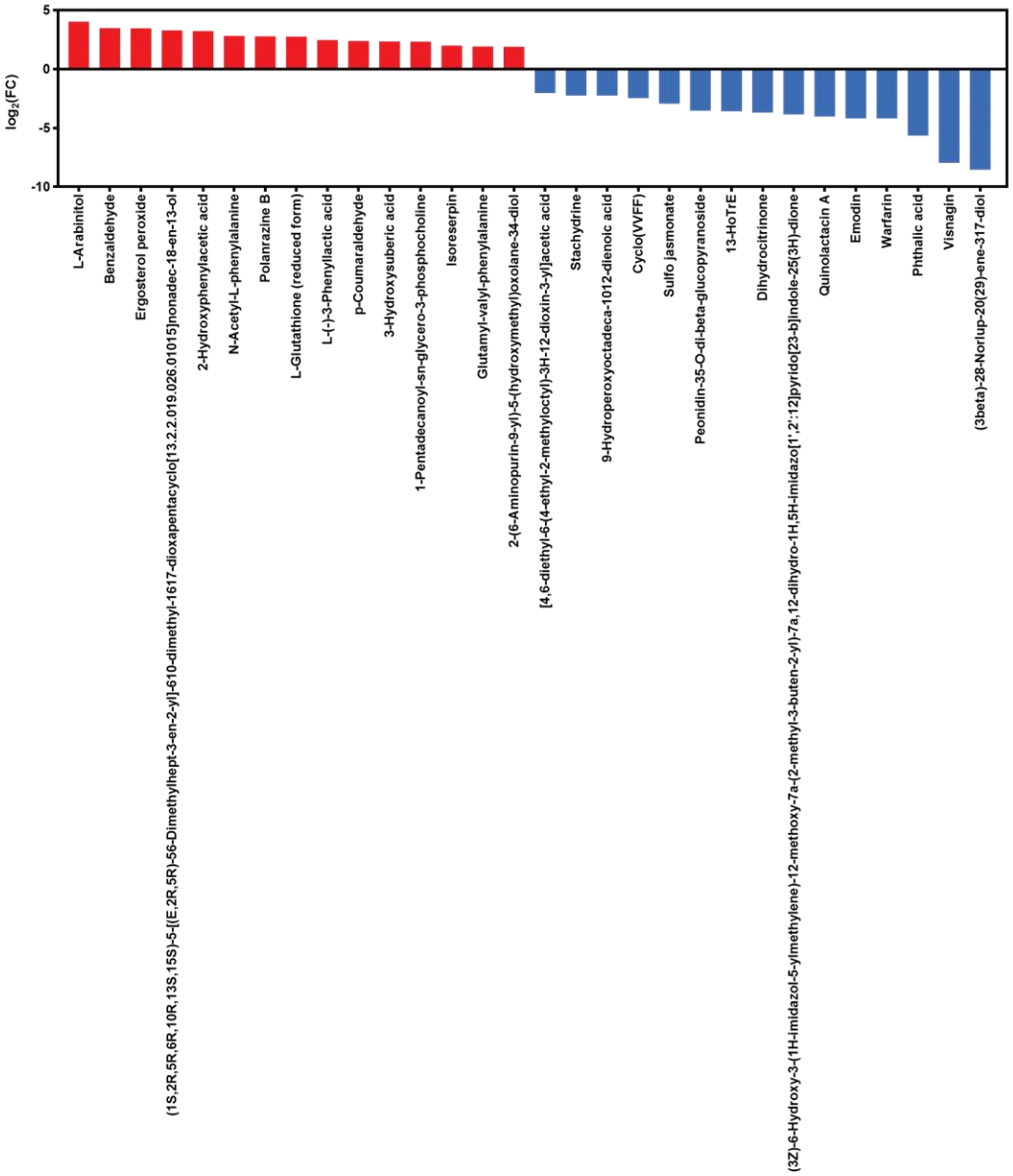

Figure S2. The top 30 differential metabolites ranked by FC values between S2 and CK groups. Metabolites with significant up-regulation were shown in red; significant down-regulation were shown in blue.
